# Leisure time physical activity among older adults in Brazil: a time series analysis of a population-based survey (2009-2020)

**DOI:** 10.1590/0102-311XEN212622

**Published:** 2023-10-09

**Authors:** Marcela Mello Soares, Thais Cristina Marquezine Caldeira, Taciana Maia de Sousa, Leandro Fórnias Machado de Rezende, Rafael Moreira Claro

**Affiliations:** 1 Universidade Federal de Minas Gerais, Belo Horizonte, Brasil.; 2 Universidade Federal de São Paulo, São Paulo, Brasil.

**Keywords:** Aging, Physical Activity, Chronic Disease, Envelhecimento, Atividade Física, Doença Crônica, Envejecimiento, Actividad Física, Enfermedad Crónica

## Abstract

The practice of leisure time physical activity brings several health benefits, such as the prevention of noncommunicable diseases. Investigating the temporal trend of physical activity practice in older adults by sociodemographic characteristics and geographical regions could be important to plan public health policies and effective interventions. This is a time series study that analyzes the temporal trend of leisure time physical activity among Brazilian older adults with data from 2009 to 2020. For this, we used a sample of 186,097 older adults (≥ 60 years old) obtained from the *Risk and Protective Factors Surveillance System for Chronic Noncomunicable Diseases Through Telephone Interview* (Vigitel) (2009-2020). Information on leisure time physical activity and sociodemographic and health characteristics were collected. Prais-Winsten regression was used to identify significant trends in the annual variation of the leisure time physical activity indicators. Practice of ≥ 150 minutes/week of moderate intensity leisure time physical activity varied from 23.3% to 27.5% (0.41p.p./year) (2009-2020), with a higher increase during 2015-2020 (0.59p.p./year). The increase in the most recent period occurred among men, aged from 60 to 69 years, with lower educational level, residing in the Northeast Region, and without self-reported chronic diseases. These results may contribute to the evaluation of Brazilian health policies targeting the leisure time physical activity practice in older adults.

## Abstract (pt)

A prática de atividade física no lazer traz diversos benefícios à saúde, como a prevenção de doenças não transmissíveis. Investigar a tendência temporal da prática de atividade física em idosos de acordo com características sociodemográficas e regiões geográficas pode ser importante para formular políticas públicas de saúde e intervenções efetivas. Este é um estudo de série temporal para analisar a tendência temporal de idosos brasileiros à prática de atividade física no lazer entre 2009 e 2020. Uma amostra de 186.097 idosos (≥ 60 anos) foi obtida do *Sistema de Vigilância de Fatores de Risco e Proteção para Doenças Crônicas por Inquérito Telefônico* (Vigitel) (2009-2020). Foram coletadas informações sobre prática de atividade física no lazer e características sociodemográficas e de saúde. A regressão de Prais-Winsten foi utilizada para identificar tendências significativas na variação anual dos indicadores de prática de atividade física no lazer. A prática de atividades físicas moderadas por ≥ 150 minutos/semana variou de 23,3% a 27,5% (0,41p.p./ano) (2009-2020) com maior aumento em 2015-2020 (0,59p.p./ano). O aumento mais recente ocorreu entre homens, indivíduos entre 60 e 69 anos de idade, aqueles com menor escolaridade, residentes na Região Nordeste e sem doença crônica autorreferida. Esses resultados podem contribuir para a avaliação das políticas de saúde brasileiras voltadas à prática de atividade física no lazer em idosos.

## Abstract (es)

La práctica de actividad física por ocio trae diversos beneficios a la salud, como la prevención de enfermedades no transmisibles. Investigar la tendencia temporal de la práctica de actividad física en adultos mayores según las características sociodemográficas y las regiones geográficas puede ser importante para formular políticas de salud pública e intervenciones efectivas. Este es un estudio de serie temporal para analizar la tendencia temporal de adultos mayores brasileños a la práctica de actividad física por ocio entre 2009 y 2020. Se obtuvo una muestra de 186,097 adultos mayores (≥ 60 años) del *Vigilancia de Factores de Riesgo y Protección para Enfermedades Crónicas No Transmisibles por Entrevista Telefónica* (Vigitel) (2009-2020). Se recopiló información sobre práctica de actividad física por ocio y las características sociodemográficas y de salud. Se utilizó la regresión de Prais-Winsten para identificar tendencias significativas en la variación anual de los indicadores de práctica de actividad física por ocio. La práctica de actividades físicas moderada por ≥ 150 minutos/semana varió de 23,3% a 27,5% (0,41p.p./año) (2009-2020) con el mayor aumento en 2015-2020 (0,59p.p./año). El aumento más reciente ocurrió entre hombres, las personas entre 60 y 69 años de edad, aquellos con menor educación, residentes en la Región Nordeste y sin enfermedad crónica autoinformada. Estos resultados pueden contribuir a la evaluación de las políticas de salud brasileñas dirigidas a la práctica de actividad física por ocio en adultos mayores.

## Introduction

Brazil has been experiencing a demographic and epidemiological transition since the 1960s ^1^, with greater aging rates than high-income countries ^2^. Data from the Brazilian Institute of Geography and Statistics (IBGE) indicates that individuals aged over 60 years are the fastest-growing segment in the Brazilian population ^3^. The population of older adults increased from 14.2 million in 2000 to 19.6 million in 2010, and estimates indicate it may reach 41.5 million by 2030 and 73.5 million by 2060 ^3^. Moreover, the improvement in healthcare access resulted in an epidemiological transition characterized by a decrease in the burden of infectious diseases and an increase of noncommunicable diseases (NCDs) ^4^, resulting mainly from unhealthy habits and lifestyle ^5^.

NCDs present a multifactorial etiology but are mostly determined by a group of four modifiable risk factors, namely: physical inactivity, unhealthy eating habits, smoking, and alcohol abuse ^6^. Monitoring these risk factors is important to identify emerging threats to the older adults population, considering the greater propensity to develop NCDs with aging ^6^ ^,^ ^7^ ^,^ ^8^.

The total physical activity performed by an individual can be obtained by the sum of activities performed in four domains: occupational activity, household activity, commuting activity, and leisure time activity ^9^. Leisure time physical activity is a component of global physical activity that includes sport participation, exercises, and others, and is different from occupational and household activities. The World Health Organization (WHO) physical activity recommendation for adults (aged 18 to 64 years) is 150-300 minutes of moderate-intensity aerobic physical activity, or engage in 75-150 minutes of vigorous-intensity physical activity per week, or an equivalent combination of moderate- and vigorous-intensity activity throughout the week, involving aerobic and muscle-strengthening activities ^10^. This recommendation is also applied to individuals aged 65 and over; however, those with debilitating conditions must be assisted by a healthcare professional ^10^.

Physical activity practice is an important factor to promote health and quality of life in the older population ^11^, being associated with primary and secondary prevention of NCDs ^9^ ^,^ ^12^. In 2018, the prevalence of older Brazilian adults that performed physical activity 3-4 days per week was 36.1% ^13^. However, to this date, no study evaluating the temporal trend of physical activity practice in older adults from Brazil or other countries was found, highlighting the importance of this study.

Considering that leisure time physical activity is an important factor for preventing NCDs and improving quality of life of the older adults, investigating the temporal trend of physical activity practice in older adults by sociodemographic characteristics and geographical regions could be important to inform and plan public health policies and population-wide interventions. This information may allow the development of effective public policies targeting this population subgroup, promoting healthy habits. Thus, we aimed to analyze the temporal trend of leisure time physical activity in older Brazilian adults in 26 Brazilian capitals and the Federal District from 2009 to 2020.

## Methods

### Study population and sampling

Data from the *Risk and Protective Factors Surveillance System for Chronic Noncomunicable Diseases Through Telephone Interview* (Vigitel) collected from 2009 to 2020 was used. Vigitel is composed of a probabilistic sample of adults (≥ 18 years) living in households with at least one landline telephone ^14^. The Vigitel sampling was conducted in two stages. The first stage consisted of screening eligible participants based on at least 10,000 landline telephones selected randomly in each city, and the second stage consisted of selecting one adult among the residents of each household (simple random sample), inviting them to participate in the survey. Approximately 2,000 participants were interviewed in each city per year, totaling more than 590,000 interviews from 2009 to 2020 ^14^. Vigitel estimates are weighted to represent the socioeconomic composition (sex, age, educational level). More details on Vigitel sampling and weighting processes are provided in the system annual report ^14^.

The analytical sample of this study involved 186,097 Brazilian older adults (≥ 60 years old) who responded to the physical activity questionnaire.

### Data collection and study variables

The interviews were conducted by a previously trained interviewer using an electronic questionnaire ^14^. The practice of leisure time physical activity was evaluated by three questions: (1) “Have you practiced sports or exercise in the last three months? (yes; no)”; (2) “How many days do you usually practice sports/exercise per week? (1-2 days/week; 3-4 days/week; 5-6 days/week; everyday (including Saturday and Sunday)”; and (3) “On these days, how much time do you spend in sports/exercise? (< 10 minutes/day; 10-19 minutes/day; 20-29 minutes/day; 30-39 minutes/day; 40-49 minutes/day; 50-59 minutes/day; ≥ 60 minutes/day)”. The weekly frequency and duration of leisure time physical activity was multiplied to obtain the time spent with leisure time physical activity per week. Based on the WHO physical activity recommendation ^9^, information regarding the intensity, weekly frequency, and usual duration of physical activity were used to construct the indicator “Sufficient leisure time physical activity (≥ 150 minutes/week) (yes; no)”. Initially, the types of physical activity were classified according to the concept of metabolic equivalents (METs) ^14^ ^,^ ^15^ to classify the intensity of physical activity as moderate (3-6 METs) or vigorous (≥ 6 METs). Then, time spent with physical activity per week was estimated multiplying average weekly frequency (days) by the average duration of the practice (minutes). Total time spent with physical activity was then multiplied by 2 in the case of those reporting vigorous activities. Finally, a dichotomous variable was created to identify who reached the recommended levels of physical activity (≥ 150 minutes/week).

Information on gender (men; women), age groups (60-69; 70-79; ≥ 80 years old), educational level (0-8; 9-11; ≥ 12 years), marital status (married or stable union; single), geographic region (Central-West; Northeast; North; Southeast; South), and self-reported chronic diseases (hypertension and/or diabetes mellitus [yes; no]) complemented the analysis.

### Data analysis

Initially, the study population was described by its distribution (%) according to sociodemographic (gender, age group, educational level, marital status, and geographic region) and health (chronic diseases) characteristics in each of the years (2009-2020). The prevalence of any leisure time physical activity in the three months prior to the Vigitel interview was estimated for the total population and according to sociodemographic and health characteristics for the same period. Weekly frequency and usual duration of practicing were analyzed by distributing the individuals, in each of the years, among five weekly frequency categories (1-2 days/week; 3-4 days/week; ≥ 5 days/week; no practice) and seven categories of usual duration of practicing(< 10 minutes/day; 10-19 minutes/day; 20-29 minutes/day; 30-39 minutes/day; 40-49 minutes/day; 50-59 minutes/day; ≥ 60 minutes/day; no practice) - the latter involving only individuals who reported no physical activity practice in the three months prior to the interview. In both cases, the categories used correspond to those already adopted in the Vigitel questionnaire ^14^. This analysis was performed for the entire population and period studied. Finally, the frequency of sufficient leisure time physical activity (≥ 150 minutes/week) was estimated for each of the years for the entire population and according to their sociodemographic and health characteristics.

Prais-Winsten regression models were used to analyze the magnitude and identify significant trends (increase and decrease) in the annual variation of the physical activity indicators described above, for the entire (2009-2020) and the most recent (2015-2020) period. These models adopted the value of the indicator (for example, the percentage of individuals who practiced moderate activity in the year) as an outcome (dependent variable) and the year of the survey as an explanatory variable (independent variable). The regression coefficient was expressed in percentage points (p.p.) in the evaluated period and indicates the average annual variation. Temporal variations of which the regression coefficients were different from zero (p ≤ 0.05) were considered significant.

The weighting adopted by the Vigitel survey was considered in all analyses. Data processing and analysis were performed using the Stata statistical program, version 16.1 (https://www.stata.com), considering the complex design of the study sample.

## Results

A total of 186,097 older adults (≥ 60 years) were interviewed by Vigitel from 2009 to 2020. This population was composed mostly by women (59.9%), aged 60 to 69 years (57.3%), from 0 to 8 years of education (66.5%), without a partner (single, separated, divorced, or widowed) (44%), residing in the capitals of the Southeast (51.5%), and without self-reported chronic disease (63.1%). From 2009 to 2020, there was an increase in the proportion of individuals aged 80 years or older (from 9.7% in 2009 to 13% in 2020 [0.29p.p./year; p ≤ 0.05]), in contrast to a decrease in the proportion of those aged from 70 to 79 years old (from 33.2% in 2009 to 29% in 2020 [-0.35p.p./year; p < 0.001]). Similarly, an increase in the proportion of individuals with 9 years or more of education was observed, concurrently with a decrease of those with 8 years or less of education (Table 1).

**Table 1:** Distribution * (%) of the older adult population (≥ 60 years) from the Brazilian capitals and the Federal District, according to gender, age group, educational level, marital status, geographic region, and chronic diseases. *Risk and Protective Factors Surveillance System for Chronic Noncomunicable Diseases Through Telephone Interview* (Vigitel), 2009-2020.

Characteristics	2009	2010	2011	2012	2013	2014	2015	2016	2017	2018	2019	2020	2009-2020 (p.p./year) **	2015-2020 (p.p./year) **
Gender														
Men	40.1	40.0	40.3	39.4	40.5	41.0	39.3	39.4	40.2	40.0	38.8	41.7	-0.02	0.11
Women	59.9	60.0	59.7	60.6	59.5	59.0	60.7	60.6	59.8	60.0	61.2	58.3	0.02	-0.11
Age group (years)														
60-69	57.1	57.7	57.8	56.2	56.0	57.8	57.6	56.8	57.5	57.5	57.9	58.0	0.07	0.19 ***
70-79	33.2	31.5	30.9	32.1	31.6	29.7	28.8	29.5	28.7	30.0	29.0	29.0	-0.35 ^#^	0.01
80 and more	9.7	10.8	11.3	11.7	12.4	12.4	13.6	13.7	13.8	12.5	13.1	13.0	0.29 ***	-0.20
Educational level (years)														
0-8	74.0	71.7	70.6	67.8	69.3	68.2	64.4	66.3	63.9	62.9	60.6	58.7	-1.22 ^#^	-1.38 ***
9-11	15.1	16.7	16.9	19.4	17.7	17.9	18.6	18.6	20.1	20.7	22.2	21.8	0.55 ^#^	0.88 ^#^
11 and more	10.9	11.7	12.4	12.8	13.0	13.9	16.9	15.1	16.0	16.4	17.2	19.5	0.68 ^#^	0.56
Marital status														
Married or stable union	43.0	43.2	43.1	42.7	43.9	41.7	44.6	42.4	41.4	39.5	39.8	40.5	-0.32 ***	-0.84 ***
Single	57.0	56.8	56.9	57.3	56.1	58.3	55.4	57.6	58.6	60.5	60.2	59.5	0.32 ***	0.84 ***
Geographic region														
Central-West	8.8	9.0	9.1	9.3	9.5	9.8	10.0	9.8	10.4	10.0	10.1	10.9	0.16 ^#^	0.12
Northeast	23.1	21.9	22.3	22.5	22.2	22.3	22.4	22.4	22.2	22.2	22.2	22.0	-0.03	-0.08 ***
North	6.8	6.7	6.8	6.9	7.0	7.1	7.0	7.1	7.2	7.3	7.3	7.1	0.05 ^#^	0.03
Southeast	51.7	53.2	52.4	52.1	51.8	51.3	51.3	51.0	50.7	51.3	51.1	50.7	-0.17 ^#^	-0.05
South	9.7	9.2	9.4	9.2	9.5	9.4	9.3	9.6	9.5	9.3	9.3	9.4	-0.01	-0.03
Chronic diseases														
Yes	36.1	36.3	36.4	36.5	36.9	35.2	38.5	34.5	37.9	37.1	38.9	37.5	0.18 ***	0.44
No	63.9	63.7	63.6	63.5	63.1	64.8	61.5	65.5	62.1	62.9	61.1	62.5	-0.18 ***	-0.44

p.p.: percentage points.

* Weighted percentage to adjust the sociodemographic distribution of the Vigitel sample to the distribution of the older adult population of each city estimated for each year of study;

** Annual variation corresponding to the Prais-Winsten regression coefficient value;

*** p ≤ 0.05;

^#^ p < 0.001.

The frequency of older adults who reported having performed leisure time physical activity in the three months prior to the interview increased significantly from 37.2% in 2009 to 48% in 2020 (0.93p.p./year; p < 0.001), with a greater increase from 2015 to 2020 (1.17p.p./year; p < 0.001) (Table 2). For the entire period studied, a greater increase was observed among men (1.14p.p./year; p < 0.001), aged 60 to 69 years old (1.10p.p./year; p < 0.001), without a partner (1.01p.p./year; p < 0.001), living in the North Region (1.40p.p./year; p < 0.001), and who did not have self-reported chronic disease (1.05p.p./year; p < 0.001). Results for the most recent period (2015-2020) were similar to those of the entire period (2009-2020), with larger annual variations (Table 2).

**Table 2:** Prevalence * (%) of the older adult population (≥ 60 years) who practiced leisure time physical activity in the three months preceding the interview by telephone survey in the Brazilian capitals and the Federal District, according to gender, age group, educational level, marital status, geographic region, and chronic diseases. *Risk and Protective Factors Surveillance System for Chronic Noncomunicable Diseases Through Telephone Interview* (Vigitel), 2009-2020.

Characteristics	2009	2010	2011	2012	2013	2014	2015	2016	2017	2018	2019	2020	2009-2020 (p.p./year) **	2015-2020 (p.p./year) **
Gender														
Men	40.4	39.9	44.3	43.3	38.8	42.5	44.0	48.6	46.4	50.6	49.7	53.7	1.14 ***	1.34 ***
Women	35.0	35.6	38.3	36.6	37.2	37.4	39.1	39.3	40.8	43.0	42.4	43.8	0.77 ***	1.02 ***
Age group (years)														
60-69	38.8	40.2	41.3	41.6	39.3	43.7	43.1	46.0	47.1	48.7	48.3	51.8	1.10 ***	1.35 ***
70-79	37.5	35.1	42.6	39.1	39.6	35.8	41.1	40.9	39.7	45.5	43.8	44.0	0.64 ^#^	0.93 ^#^
80 and more	26.4	28.1	32.3	28.3	26.5	28.6	32.2	35.0	33.1	35.1	34.6	39.6	0.99 ***	0.76 ^#^
Educational level (years)														
0-8	31.2	31.8	34.6	32.9	31.8	31.8	32.2	36.7	36.1	39.2	37.7	40.8	0.79 ^#^	1.22 ^#^
9-11	48.7	43.7	49.8	45.0	46.5	47.2	48.4	49.8	49.4	51.2	50.1	51.4	0.50 ***	0.44 ^#^
11 and more	61.5	62.0	62.9	64.0	58.3	67.0	66.4	62.1	62.6	65.7	65.3	65.6	0.34 ^#^	0.34
Marital status														
Married or stable union	33.4	35.3	37.1	36.6	36.6	34.8	37.0	39.5	39.0	42.5	40.0	43.4	0.74 ***	0.91 ***
Single	40.0	38.8	43.4	41.2	38.8	42.8	44.3	45.6	45.9	48.4	48.6	51.1	1.01 ***	1.26 ***
Geographic region														
Central-West	46.4	46.0	46.1	43.8	45.8	44.3	50.1	50.5	54.4	51.7	54.9	56.0	1.02 ***	1.13 ^#^
Northeast	32.5	35.4	36.2	39.0	38.9	38.3	36.7	40.6	41.7	44.9	42.3	47.2	1.04 ***	1.56 ^#^
North	27.1	33.8	33.7	37.2	32.8	33.4	39.2	41.0	41.6	44.9	43.3	43.9	1.40 ***	0.97 ^#^
Southeast	37.7	35.4	41.8	37.9	35.0	38.5	40.4	41.9	40.1	44.7	43.7	46.0	0.77 ***	1.05 ^#^
South	44.4	46.4	45.1	44.5	46.8	47.1	46.8	48.0	50.4	51.3	51.3	54.4	0.82 ***	1.33 ***
Chronic diseases														
Yes	34.9	35.4	39.3	38.1	35.6	38.0	38.4	40.2	40.3	43.7	42.3	45.5	0.82 ***	1.19 ***
No	41.2	40.7	43.2	41.1	41.7	42.2	45.3	48.3	47.5	50.0	49.7	52.1	1.05 ***	1.04 ***
Total	37.2	37.3	40.7	39.2	37.8	39.5	41.0	43.0	43.0	46.1	45.2	48.0	0.93 ***	1.17 ***

p.p.: percentage points.

* Weighted percentage to adjust the sociodemographic distribution of the Vigitel sample to the distribution of the older adult population of each city estimated for each year of study;

** Annual variation corresponding to the Prais-Winsten regression coefficient value;

*** p < 0.001;

^#^ p ≤ 0.05.

During the entire studied period, more than half of the population reported not practicing leisure time physical activity (60.3%). However, there was a reduction in this percentage over time, from 64.2% in 2009 to 55.3% in 2020 (-0.85p.p./year; p ≤ 0.001). In contrast, we observed an increase in the percentage of those who reported performing activities 1 to 2 days (from 9% to 13.5% [0.43p.p./year; p < 0.001]) and 3 to 4 days per week (12.2% to 16% [0.41p.p./year; p < 0.001]) (Table 3). Approximately one in five older adults who practiced leisure time physical activity in the three months prior to the interview reported a usual duration of practicing of 60 minutes or more (20.8%). The frequency of older adults in this usual duration of practice increased from 17.2% in 2009 to 24.8% in 2020 (0.79p.p./year; p < 0.001), and there was also an increase in the usual duration of practice from 40 to 49 minutes, from 5.7% in 2009 to 7% in 2020 (0.07p.p./year; p ≤ 0.05) (Table 3).

As a result, there was an increase in the percentage of older adults reaching the recommended level of leisure time physical activity (150 minutes/week of moderate activity or equivalent), from 23.2% in 2009 to 27.5% in 2020 (0.41p.p./year; p ≤ 0.001). This increase occurred, particularly, among men (0.64p.p./year; p < 0.001), aged 60 to 69 years (0.54p.p./year; p ≤ 0.001), with up to 8 years of education (0.34p.p./year; p < 0.001), with a partner (0.40p.p./year; p < 0.001), residing in the capitals of the North Region (0.83p.p./year; p ≤ 0.001), and with self-reported chronic disease (0.40p.p./year; p ≤ 0.001) (Table 4).

**Table 3:** Prevalence * (%) of physical activity indicators among the older adult population (≥ 60 years) from the Brazilian capitals and the Federal District. *Risk and Protective Factors Surveillance System for Chronic Noncomunicable Diseases Through Telephone Interview* (Vigitel), 2009-2020.

Indicators	2009	2010	2011	2012	2013	2014	2015	2016	2017	2018	2019	2020	2009-2020 (p.p./year) **	2015-2020 (p.p./year) **
Weekly frequency (days)														
1-2	9.0	9.1	10.2	10.1	10.0	10.5	11.4	13.0	11.1	13.1	13.3	13.5	0.43 ***	0.37 ^#^
3-4	12.2	12.0	12.7	12.6	12.9	14.3	14.3	14.3	14.7	15.9	16.3	16.0	0.41 ***	0.46 ^#^
≥ 5	14.7	14.2	15.3	15.0	14.0	13.4	14.3	14.5	14.7	15.2	13.5	15.3	0.00	-0.01
No practice	64.2	64.8	61.9	62.2	63.1	61.7	60.0	58.3	59.4	55.8	56.9	55.3	-0.85 ***	-0.87 ***
Daily duration (minutes)														
< 10	0.4	0.2	0.4	0.2	0.4	0.2	0.4	0.5	0.2	0.2	0.4	0.3	0.00	-0.03
10-19	1.3	1.5	1.7	1.4	1.5	1.4	1.2	1.2	1.7	1.5	1.6	1.5	0.01	0.07
20-29	1.6	2.1	1.7	2.2	2.0	2.0	2.1	2.2	1.6	2.2	1.8	2.4	0.01	-0.01
30-39	6.7	5.9	6.4	6.1	5.2	6.0	6.0	7.6	5.3	6.3	6.1	6.0	-0.01	-0.16
40-49	5.7	6.1	6.0	6.1	6.2	6.5	6.1	6.9	5.6	6.3	6.9	7.0	0.07 ^#^	0.14
50-59	2.9	2.2	3.4	2.9	3.1	2.5	3.0	2.6	2.5	1.7	2.3	2.8	-0.05	-0.09
> 60	17.2	17.3	18.6	18.8	18.5	19.6	21.2	20.7	23.6	26.1	24.1	24.8	0.79 ***	0.88
No practice	64.2	64.8	61.9	62.2	63.1	61.7	60.0	58.3	59.4	55.8	56.9	55.3	-0.85 ***	-0.87 ***

p.p.: percentage points.

* Weighted percentage to adjust the sociodemographic distribution of the Vigitel sample to the distribution of the older adult population of each city estimated for each year of study;

** Annual variation corresponding to the Prais-Winsten regression coefficient value;

*** p ≤ 0.001;

^#^ p ≤ 0.05.

**Table 4:** Prevalence * (%) of the older adult population (≥ 60 years) who practiced sufficient leisure time physical activity (150 minutes/week) from the Brazilian capitals and the Federal District, according to gender, age group, educational level, marital status, geographic region, and chronic diseases. *Risk and Protective Factors Surveillance System for Chronic Noncomunicable Diseases Through Telephone Interview* (Vigitel), 2009-2020.

Characteristics	2009	2010	2011	2012	2013	2014	2015	2016	2017	2018	2019	2020	2009-2020 (p.p./year) **	2015-2020 (p.p./year) **
Gender														
Men	27.3	25.6	28.2	28.3	26.6	26.2	30.2	31.1	29.2	33.2	31.7	33.7	0.64 ***	0.66 ^#^
Women	20.6	20.8	21.5	21.1	21.8	23.7	22.0	20.2	23.5	23.7	23.3	23.1	0.25 ^#^	0.50
Age group (years)														
60-69	24.7	25.8	26.6	26.4	25.9	28.4	27.4	27.1	29.1	30.8	29.7	31.6	0.54 ***	0.90 ^#^
70-79	22.9	20.0	23.2	23.1	23.7	21.5	24.2	22.6	23.5	25.4	25.2	24.0	0.30 ***	0.30
80 and more	16.0	14.2	14.7	14.5	14.2	15.4	18.3	17.7	16.8	17.3	15.7	17.1	0.22	-0.42 ^#^
Educational level (years)														
0-8	17.9	18.7	19.3	19.1	19.7	18.5	18.9	20.0	20.4	22.7	21.3	21.7	0.34 ***	0.58 ^#^
9-11	34.1	27.1	31.2	27.7	29.0	32.1	30.4	30.3	30.8	31.3	30.0	30.9	0.08	0.03
11 and more	44.6	40.7	42.5	44.0	38.1	45.8	43.4	37.1	40.9	41.2	40.9	41.3	-0.21	0.24
Marital status														
Married or stable union	19.4	19.2	20.2	21.5	20.3	21.2	21.8	20.9	22.5	24.2	22.7	23.6	0.40 ***	0.49 ^#^
Single	26.2	25.4	27.3	25.7	26.4	27.2	28.0	27.1	28.1	29.7	29.1	30.2	0.38 ***	0.57 ^#^
Geographic region														
Central-West	29.2	32.4	27.4	28.4	30.3	28.4	34.7	31.4	35.0	33.0	34.1	32.8	0.50 ^#^	0.08
Northeast	22.1	21.7	23.2	24.9	25.8	24.6	22.6	24.4	26.0	27.4	27.4	28.9	0.56 ^#^	1.20 ***
North	17.7	19.2	18.7	22.7	20.2	21.0	25.2	24.5	25.6	28.1	25.6	24.9	0.83 ***	0.13
Southeast	23.1	21.1	24.6	22.2	21.5	24.2	24.3	23.1	23.5	26.3	24.7	25.8	0.31 ^#^	0.49
South	25.5	27.6	25.7	27.7	27.4	27.2	26.6	25.3	27.6	27.7	27.5	30.1	0.18	0.68 ^#^
Chronic diseases														
Yes	20.3	20.3	22.6	22.6	22.1	23.2	23.5	23.0	23.7	25.3	23.7	25.7	0.40 ***	0.40 ^#^
No	28.6	26.8	27.1	26.3	26.6	27.5	27.9	27.3	29.2	31.3	31.1	30.6	0.30	0.78 ^#^
Total	23.3	22.7	24.2	23.9	23.8	24.7	25.2	24.5	25.8	27.5	26.6	27.5	0.41 ***	0.59 ^#^

p.p.: percentage points.

* Weighted percentage to adjust the sociodemographic distribution of the Vigitel sample to the distribution of the older adult population of each city estimated for each year of study;

** Annual variation corresponding to the Prais-Winsten regression coefficient value;

*** p ≤ 0.001;

^#^ p ≤ 0.05.

## Discussion

We conducted a time series study to analyze the temporal trend of leisure time physical activity among Brazilian older adults based on the systematic collection of data conducted by Vigitel from 2009 to 2020 that included more than 180,000 older adults from 26 Brazilian capitals and the Federal District. During the entire period studied, there was a significant increase in the proportion of older adults who reported having practiced leisure time physical activity in the three months before the study (0.93p.p./year), representing a positive evolution of this behavior in the studied population. The weekly frequency of physical activity also evolved favorably, with a reduction in the percentage of older adults who did not perform leisure time physical activity and an increase of those who practiced 1 to 4 times per week (1-2 days/week: 0.43p.p./year; 3-4 days/week: 0.41p.p./year). As for the duration of activities, there was an increase in the percentage of older adults who reported practicing activity for 40 to 49 minutes (0.07p.p./year) and 60 minutes or more (0.79p.p./year). Also, the practice of leisure time physical activity (≥ 150 minutes/week of moderate intensity or equivalent) increased from 23.3% in 2009 to 27.5% in 2020 (0.41p.p./year), with a higher increase during the most recent period (2015-2020).

Despite the favorable evolution of indicators related to the practice of leisure time physical activity, it should be noted that the scenario of the older Brazilian adults remains less than ideal. The results showed that, in the last year of the studied period (2020), over half the population (55.3%) had reported not practicing any physical activity in the three months before the interview. Despite considering the increase of the population reaching the recommended levels of physical activity, inequalities related to gender, age, education level, and geographic region, already observed in 2009, remained existing after 12 years. We highlight that these inequalities are not specific to the older adult population, also being observed in other studies with Brazilian adults ^16^ ^,^ ^17^ ^,^ ^18^. Robust evidence supports that a higher level of leisure time physical activity is frequently observed among men, younger individuals, and those with higher education level ^19^. The hypotheses behind this evidence involves cultural factors, namely: men participating more of collective activities, whereas women, prefer individual activities with less physical strength ^20^; lack of motivation to practice physical activity due to natural reduction of conditioning, especially from the second half of life ^21^ ^,^ ^22^; and practice of physical activity in other domains (occupational activity, commuting activity ^23^, and household activity) competing with leisure time physical activity among individuals with a lower education level ^24^. Adults with lower educational level are more likely to have lower socioeconomic status, leading to worse health outcomes compared to those with a higher educational level, a concept called educational gradient in health ^25^ ^,^ ^26^. To date, the extent of how socioeconomic inequalities affect physical activity among adults and how it has changed over time in Brazil is uncertain. Therefore, the monitoring of health inequalities is essential to inform and plan effective policies, programs, and actions to improve the health of the population ^4^.

Our results indicate that leisure time physical activity practice rates among the less favored groups has improved since 2009, although gender inequalities among older adults have increased and the proportion of age groups has remained similar. We observed a decrease in the difference between education levels and the geographic regions. Despite being impossible to identify the cause of this phenomenon in our results, it can be attributed, at least partly, to the improvement of health access for older adults, allowing greater access to information regarding the importance of physical activity, and to the policies and programs that promote the practice of physical activity in the recent decades.

A significant number of sedentary older adults started to practice physical activity in the 26 Brazilian capitals and the Federal District during the studied period. To the best of our knowledge, no Brazilian studies specifically addressing the motivation of the older adults to practice leisure time physical activity have been conducted. However, data from the *Brazilian National Household Sample Survey* (PNAD), conducted with individuals from Brazilian urban regions aged ≥ 15 years, suggests that the main motivations for the practice of physical activity include “relaxation and fun” (28.9%), “improvement of quality of life and well-being” (26.8%), “improvement or maintenance of physical performance” (19.9%), and “medical indication” (4.9%) ^27^. These motivations may also apply to older adults.

The analysis stratified by the presence of self-reported NCDs also provides important information regarding the practice of leisure time physical activity by older adults. In 2009, 36.1% of the older adults had chronic disease (diabetes and/or hypertension), and this percentage has increased to 37.5% in 2020. A higher prevalence of leisure time physical activity was observed among individuals without self-reported NCDs. It is known that this scenario might be associated to the greater presence of disabling conditions among individuals with chronic disease ^28^, but that alone is not enough to explain all the difference observed. This finding might indicate that unhealthy behaviors, considered to be risk factors for these diseases, such as physical inactivity, tend to persist after their diagnosis and that these individuals are less susceptible to adhere current actions to promote physical activity.

Although the prevalence of older adults with the recommended level of physical activity is still less than ideal, the trend of increase observed is a positive result, and the same scenario was observed in higher income countries, such as the United States ^28^. A population-based study that analyzed data from three leading surveillance systems in the United States (*National Health and Nutrition Examination Survey* - NHANES, *Behavioral Risk Factor Surveillance System* - BRFSS, and *National Health Interview Survey* - NHIS) reported a prevalence ranging from 27% to 44% of older adults with a recommended level of physical activity, with an increasing trend from 1998 to 2013 ^28^. According to the authors, such increase is positive but there is still room for improvement, considering that over half of the older population does not reach the adequate level of physical activity, and this outcomes may also apply to the scenario of Brazilian older adults presented in our study.

Our study did not aim to explain the causes of the trends in physical activity; however, it is plausible to assume that they could be partially explained by Brazilian public policies conducted during the period, such as the Health Gym Program ^29^, and the Live Healthy Program ^30^. However, until this moment, no studies on the efficacy of these actions were found to allow the understanding of their real impact on the prevalence of physical activity among older Brazilian adults. Possibly, actions with lower costs became an interesting option for public health managers in recent years, such as the development of guides for the physical activity practice among the Brazilian population, including older adults, as a form of active aging promotion ^29^ ^,^ ^30^ ^,^ ^31^.

Our study presents some limitations that must be addressed. Firstly, Vigitel is a survey conducted using telephone interviews. Therefore, the practice of leisure time physical activity was self-reported and, thus, prone to measuring errors. However, self-reported information is often used in large surveys on health conditions and lifestyle due to the facility and the low application cost in large population samples ^27^ ^,^ ^32^ ^,^ ^33^ ^,^ ^34^. Even though the use of this self-reported information may result in measuring errors, it is plausible to assume that this limitation is constant over time, so the identified trends, or even the annual variation, were not completely affected. Finally, the Vigitel sample was exclusively composed of individuals who have a landline telephone in a Brazilian capital city, a characteristic inherent to the methodology of the survey, which represents a potential risk to the representativeness of the sample, reducing the participation of some population groups, such as men and low-income individuals. We highlight, however, that Vigitel uses adequate weighting factors to adjust the estimates and correct the differences between the population with and without a landline telephone, allowing the extrapolation of the results to the total Brazilian population ^31^.

## Conclusion

Our results showed an increasing temporal trend in the prevalence of leisure time physical activity among older adult population in the 26 Brazilian capitals and the Federal District, especially among younger older adults, with a lower education level, residents of the capitals in the North Region, and without self-reported chronic diseases. The weekly frequency of physical activity also evolved favorably, with a reduction in the percentage of older adults who did not perform leisure time physical activity and an increase of those who practiced from 1 to 4 days per week. This study present unprecedented data that may be applied in the evaluation of health policies and programs or even in the proposal of new actions targeting the leisure time physical activity practice by older adults.
